# Metformin Alleviates Endometriosis and Potentiates Endometrial Receptivity *via* Decreasing VEGF and MMP9 and Increasing Leukemia Inhibitor Factor and HOXA10

**DOI:** 10.3389/fphar.2022.750208

**Published:** 2022-02-22

**Authors:** Jing Cheng, Chunyang Li, Yingfen Ying, Jieqiang Lv, Xianqin Qu, Eileen McGowan, Yiguang Lin, Xueqiong Zhu

**Affiliations:** ^1^ Department of Obstetrics and Gynaecology, The Second Affiliated Hospital and Yuying Children’s Hospital of Wenzhou Medical University, Wenzhou, China; ^2^ School of Life Sciences, University of Technology Sydney, Sydney, NSW, Australia; ^3^ Department of Biochemistry, School of Basic Sciences, Wenzhou Medical University, Wenzhou, China

**Keywords:** endometriosis, metformin, leukemia inhibitor factor, HOXA10, endometrial receptivity, vascular endothelial growth factor, matrix metalloproteinase-9

## Abstract

**Background:** Endometriosis affects endometrial receptivity, a key factor for successful embryo implantation. Metformin treatment is associated with alleviating the symptoms of endometriosis; however the mechanism of metformin action is unclear. Neoangiogenesis plays an important role in the development and recurrence of endometriosis. In addition, the leukemia inhibitor factor (LIF) and HOXA10 genes are also distinguishing markers of endometriosis (decrease) and endometrial receptivity (increase). This study investigated the therapeutic potentials of metformin and the underlying mechanism using an *in vivo* rat endometriosis model.

**Methods:** Female Wistar albino mature rats with experimentally induced endometriosis were used in this study. Metformin was administered at doses of 100 mg/kg/d and 200 mg/kg/d. The volume of endometriotic implants was assessed. The protein and mRNA expression of the vascular endothelial growth factor (VEGF), matrix metalloproteinase-9 (MMP-9), the endometrial receptivity markers, LIF and HOXA10, were measured in the endometrium of rats with endometriosis.

**Results:** Metformin treatment significantly suppressed the growth of endometriotic implants. Further, the expression of VEGF and MMP-9 protein and mRNA in endometriotic implants were significantly reduced. Metformin also significantly upregulated LIF and HOXA10 expression in endometrium from rats with endometriosis. The inhibitory effect of metformin on the growth of endometriotic implants, VEGF and MMP-9, and upregulating effect on LIF and HOXA10, was optimal at a dose of 100 mg/kg/d.

**Conclusion:** Our *in vivo* data demonstrates that metformin treatment alleviates endometriosis and potentiates endometrial receptivity. The underlying mechanisms are associated with decreased expression of VEGF and MMP-9 genes and upregulation of the LIF and HOXA10 genes. The effect of metformin was optimal at 100 mg/kg/d. These findings provide a potential alternative for women with endometriosis with the potential to increase fertility. Metformin is an approved drug by FDA for diabetes and this study may add another potential clinical use for metformin.

## Introduction

Endometriosis is a common disorder among women of reproductive age and is a major contributor to pelvic pain and infertility. Endometriosis affects approximately 10–15% of women of reproductive age (Giudice, 2010; Dmitrieva et al., 2014; Sarria-Santamera et al., 2020; Rowlands et al., 2021). Neoangiogenesis plays an important role in the development and recurrence of endometriosis, as angiogenic stimuli provides the blood flow required for implantation and enables endometrial cells to attach and grow on the mesothelial surface. Angiogenesis is mainly mediated by the vascular endothelial growth factor (VEGF) and its’ receptor, VEGFR. Recently, VEGF has been indicated as an independent biomarker for endometriosis (O et al., 2019) Endometriotic lesions and surrounding tissue are highly vascularized and therefore inhibition of angiogenesis has been proposed as a novel therapeutic option for endometriosis. Matrix metalloproteinases (MMPs) are hypothesised to play a role in ectopic implantation and invasion of endometrium tissue (Aresu et al., 2012). One important member of the MMPs family, MMP-9, is known to participate in both invasion and metastasis of various tumours, and potentially plays a crucial role in both occurrence and progression of endometriosis (Machado et al., 2016; Kim et al., 2017). Approximately 35–50% of endometriosis patients experience infertility, while 25–50% of infertile women have endometriosis (Sensky and Liu, 1980). Two mechanisms proposed to explain the detrimental influence of endometriosis on fertility are poor quality of oocytes and embryos with defective implantation ability (Bulletti et al., 2005).

There are a few biomolecular pathways that regulate implantation and endometrial receptivity. Leukemia inhibitor factor (LIF) is the most pleiotropic member of the interleukin-6 family of cytokines and has paradoxically opposing effects, including both stimulating and inhibiting cell proliferation, and differentiation and/or survival (Nicola and Babon, 2015). In the endometrium, LIF is expressed in a menstrual cycle-dependent manner, with the highest level of LIF expression occurring at the time of implantation (Lass et al., 2001), suggesting LIF assessment can be used as a predictor of reproductive success (Mikolajczyk et al., 2007). Reduced endometrial LIF expression is strongly associated with poor reproductive outcome in mice (Stewart et al., 1992; Charnock-Jones et al., 1994; Franasiak et al., 2014). Importantly, LIF expression has been found to be lower in women suffering from endometriosis (Schmitz et al., 2017), suggesting that LIF expression is a vital marker of infertility in women with endometriosis (Dimitriadis et al., 2006; Alizadeh et al., 2011). HOXA10 is a transcription factor that is crucial for the development and patterning of the uterus during embryogenesis. Sex steroids and embryos drive expression of HOXA10, with peak expression occurring at the time of implantation in response to rising progesterone levels (Sarno et al., 2005; van Mourik et al., 2009; Zhao et al., 2014; Chen et al., 2016). Decreased expression of HOXA10 in the endometrium during the window of implantation has been found in women with endometriosis (Taylor et al., 1999). Taken together, the expression of LIF and HOXA10 in the endometrium may affect receptivity of endometrium in women with endometriosis.

Currently, surgery, gonadotropin-releasing hormone (GnRH) agonists, oral contraceptive pills, progestins, and nonsteroidal anti-infiammatory drugs are mainstream therapeutic options for endometriosis (Dunselman et al., 2014; Collinet et al., 2018; Falcone and Flyckt, 2018; Schwartz et al., 2020). Estrogen is the key driver of endometriosis lesion development and as such, the majority of established therapies targeting the estrogen pathway, creating a hypoestrogenic state to prevent relapse and offering temporary relief. However, decrease in estrogen levels affects endometrium epithelial proliferation leading to failure of embryo implantation (Xu et al., 2013) thus, women are unable to conceive during treatment for endometriosis. Metformin, originally used as a first line treatment for type 2 diabetes, is now a widely used treatment for women with polycystic ovary syndrome (PCOS) (Fraison et al., 2020; Guan et al., 2020; Kim et al., 2020). It has been reported that metformin treatment reverses endometriotic implants in a rat model of endometriosis (Oner et al., 2010), through increasing superoxide dismutase activity and tissue inhibitor of MMP-2, and decreasing VEGF expression and MMP-9 (Yilmaz et al., 2010). However, the effect of metformin on endometriosis relevant to infertility remains to be clarified. In particular, the regulatory role of metformin in the implantation and endometrial receptivity through LIF and HOXA10 expression in endometrium is unclear.

Thus, the aims of this study were to first to examine VEGF and MMP-9 expression from endometriotic implants in rats with endometriosis and second to investigate the effects of metformin on endometrial receptivity through regulation of LIF and HOXA10 expression. This study will thereby identify molecular pathways that can be exploited for endometriosis therapies and potentially increase fertility in women with endometriosis.

## Materials and Methods

### Animals

Thirty-two female Wistar albino mature rats aged 8–10 weeks, weighing between180–220 g, were used to study the induction of endometriosis. The rats were purchased from the Laboratory Animal Centre, Wenzhou Medical University. Rats were caged in a controlled environment of 22 ± 2°C with 12-h light/dark cycles. All rats were observed for 1 week to ascertain health before surgery. The Laboratory Animal Ethics Committee of Wenzhou Medical University approved this study (ethics number: wydw 2015-0458).

### Endometriosis Model and Treatment

Endometriosis was surgically induced according to the method described previously (Oner et al., 2010). In brief, after anesthetized and abdomen opening, one uterine horn was ligated and removed. The excised horn was bisected along its macro-axis, and 5 mm × 5 mm sections dissected. These explants were then anchored onto the flank inside the abdominal wall with the endometrial surface facing the peritoneum. The development of endometriosis was determined by measuring the surface area (length × width × height in millimetres) 21 days after surgical procedure. Rats with endometriosis were randomly divided into three groups and treated by oral gavage with either vehicle (saline) or metformin (Santa Cruz, CA, United States) at two different concentrations. Group 1 (control group, *n* = 10) was treated with saline (4 ml/kg/day), group 2 was treated with metformin 100 mg/kg/day (ML, *n* = 11) and group 3 were treated with 200 mg/kg/d of metformin (MH, *n* = 11).

The metformin treatment dosages used in this study were similar to those described previously (Oner et al., 2010). In a clinical setting, metformin is prescribed at a dosage of 1,000 to 2,000 mg/d for women diagnosed with PCOS and insulin resistance. Thus, to correlate these dosages in a rat model, we used the conversion equation, described in (Nair and Jacob, 2016) and determined our experimental treatment dosages to be 100 and 200 mg/kg/d for rats with endometriosis.

The endometriotic implant samples from the abdominal wall and endometrium samples from the uterus were collected from rats at diestrus II stage, as described previously (Marcondes et al., 2002). Briefly, following 6 weeks of treatment, rats at diestrus II were confirmed by increased lymphocytes in vaginal smears. Rats were anaesthetized via inhalation of chloral hydrate and subsequently subjected to a laparotomy to excise the uterus endometrium as well as the endometriotic implants. The samples were immediately stored in liquid nitrogen or formalin for future analysis. For rats where the estrous cycle was not clearly identified, samples were collected for 3 days post 6 weeks of treatment and metformin treatment was continued for a further 3 days.

**FIGURE 1:**
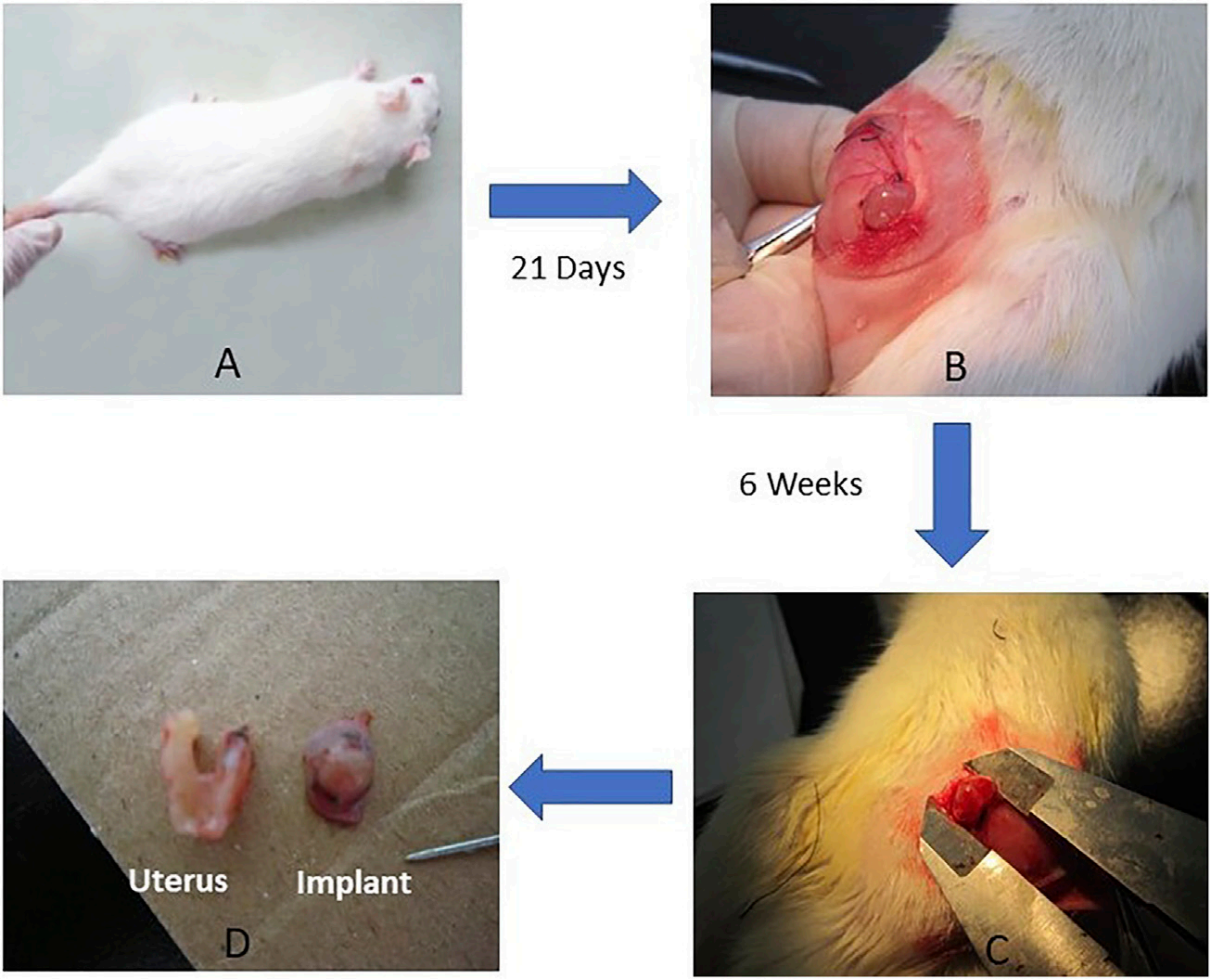
Preparation of endometriosis rat model. **(A)** Wistar Female Mature Rats were used to surgically induce the endometriosis model (*n* = 35). The surgery included removing a uterus horn and then anchoring to abdominal wall (uterus implant). **(B)** 21 days after the surgical procedure, model confirmation was done by measuring the surface area of the implant. **(C)** At the end of 6 weeks’ treatment, the size of implant was measured, and tissues (uterus and implant) were harvested **(D)** for efficacy assessment and mechanism investigation.

The key steps in the preparation of the endometriosis rat model and experimental design are summarised in Figure 1.

### Endometrial Implant Assessment

To evaluate the effect of metformin on the development of endometriotic implants, the obtained endometrium and endometriotic implants were measured for length, width, and height. The measurement procedure was performed by a researcher who was blinded to the study.

### Immunohistochemistry

The endometrium and implant tissue from rats were excised and immediately fixed in 10% buffered formalin for 24 h. After de-waxing through increasing grades of ethanol and rendered transparent using Xylene, the formalin-fixed endometriotic implants and endometrium sections were embedded in formalin blocks and sectioned at 4 µm thickness. Slides were de-waxed through descending grades of ethanol to distilled water and through xylol deparaffinization. Endogenous peroxidase was blocked with 3% H_2_O_2_ for 10 min then washed in PBS. The slides were pretreated with citrate buffer (pH 6.0) and incubated at 90°C for 20 min and washed again with PBS. The tissues were incubated with the appropriate primary antibody overnight at 4°C. Goat anti-LIF polyclonal antibody (Santa Cruz, B2813) was used at a dilution of 1:30. A rabbit anti-HOXA-10 polyclonal antibody (Bios, YSLS25W) was used at a dilution of 1:50; A rabbit anti-MMP9 polyclonal antibody (abcam GR149385-3) was used at a dilution 1:60. A mouse anti-VEGF polyclonal antibody (abcam, GR145343-1) was used at a dilution 1:100. The secondary antibody was then incubated for 30 min and subsequently tissues were incubated with avidin and biotinylated peroxidase for a further 15 min. To visualize the staining, the tissues were incubated with 3, 3′-diaminobenzidine (DAB) for 5 min. Hematoxylin and Eosin (H&E) staining was used for counterstaining. Finally, tissues were sealed with resin. Negative controls were evaluated on additional sections by omitting the primary antibodies. All tissues were evaluated with a light microscope (Olympus BX43; Olympus Optical, Tokyo, Japan). Three random fields on each slide at ×200 magnifications were used to evaluate the samples. All tissues were evaluated by two blinded histologists. IHC results were further evaluated by a semiquantitative approach to assign H-scores for endometrium samples and endometriotic implants. Immunoreactivity was evaluated with a H-score in an area of approximately 100 cells. The staining intensity was first determined for each cell in a fixed field and the average intensity of the entire field of view, corresponding to the presence of negative, weak, intermediate, and strong staining, was allocated a score from 0 to 3. Samples were then further evaluated; the percentage of positively stained cells within the field of view was assigned 1 of 4 categories (1%–25%,26%–50%,51%–75%,76%–100%). The H-scores (*H*) for VEGF, MMP-9, LIF and *HOXA10* were calculated with formula H=∑Pi , where *i* is the intensity, and P is the percentage of positively stained cells.

### Quantitative RT-PCR

Expression of HOXA10 and LIF mRNAs in the endometrium of the rat model of endometriosis were evaluated using quantitative real time polymerase chain reaction (qRT-PCR). To extract total RNA, 100 mg of each tissue sample was homogenised in TRIzol Reagent (Invitrogen, Carlsbad, CA) and incubated for 5 min. Chloroform 0.2 ml was added to the homogenate and samples incubated for a further 3 min before centrifugation at 12,000 *g* for 15 min at 4°C. The clear aqueous phase was transferred to a fresh tube. RNA precipitation was performed by mixing the sample with cold isopropyl alcohol, incubation for 10 min, and centrifugation at 12,000 *g* for a further 10 min at 4°C. Following centrifugation, RNA samples were washed twice with 75% ethanol. The RNA pellets were air-dried and resuspended with RNase-free water. Finally, RNA was stored at −80°C until RT-PCR was performed. Reverse transcription was performed using the RevertAid First Strand cDNA Synthesis kit (Fermentas Life Sciences; MA, United States) according to the manufacturer’s instructions. RT-PCR was performed using the CFX connection Real-Time PCR System with the software BIO-RAD CFX Manager. Reaction conditions included cDNA template, each primer, water, and the IQ SYBR Green Supermix for a final reaction volume of 20 μL. The sequences of all primers used are: LIF forward primer, CCC​TCT​TTA​TTT​CCT​ATT​AC; LIF reverse primer, GTA​GTC​GCA​TTG​AGT​TTG​AT; HOXA10 forward primer, CTC​CTA​CTC​CTC​CAA​CCT​GC; HOXA10 reverse primer, GTT​CCT​GCC​CAC​CGT​GCT​AT; GADPH forward primer GTG​CTG​AGT​ATG​TCG​TGG​AG; GADPH reverse primer GTC​TTC​TGA​GTG​GCA​GTG​AT. The HOXA-10 and LIF RT-PCR reactions were subjected to the following cycling parameters: 50°C for 3 min, 1 cycle; 95°C for 15 min, 1 cycle; 95°C for 10 s, 40 cycles; 59°C for 30 s, 1 cycle. Melting curve analysis was conducted to determine the specificity of the amplified products and to insure the absence of primer-dimer formation. All products obtained yielded the predicted melting temperature. Samples were run in triplicate and included negative controls. The mRNA level of each sample was normalized to that of the GADPH mRNA level. Relative gene expression data was presented as 2^−ΔΔCt^ method, and ΔΔCt for each sample refers to ratio between target gene and the control group. The results of quantitative RT-PCR were expressed as relative fold values.

### Protein Extraction and Western Blotting

One hundred mg of tissue was homogenized with the appropriate volume of lysis buffer using an ultrasonic homogenizer. The homogenates were centrifuged at 3,000 *g* for 10 min at 4°C, and the supernatant was collected. Extracted protein (30 μg) was separated using 10% sodium dodecyl sulfate-polyacrylamide gel electrophoresis (SDS-PAGE) and transferred to a methanol-activated polyvinylidene difluoride (PVDF) membrane (Millipore, Bedford, MA, United States). Membranes were blocked in 5% (w/v) skim milk/Tris buffered saline (TBS) for 1 h prior to incubation with the primary antibody. Dilution for LIF, 1:1,000; for HOXA10, 1:1,000; for MMP-9 1:1,000; for VEGF1: 1,000; for β-tubulin1:1,000 or for GADPH 1:2000. Membranes were washed three times with TBS-Tween 20 (TBST), prior to the HRP-secondary antibody (Santa Cruz, CA) incubation at 1:2500 dilutions for 1 h. Membranes were then developed using enhanced chemiluminescence (ECL) (Pierce, Illinois, United States) according to the manufacturer’s instructions and visualized using the ChemiDoc XRS + System (Bio-Rad). Statistical analysis was carried out using the ImageJ software (National Institute of Mental Health, United States) and GraphPad Prism (version 7.03) (California, United States).

### Statistical Analysis

The data are expressed as the Mean ± SD. Comparisons across the three groups were performed using one-way analysis of variance (ANOVA) followed by Dunn’s test to determine significant differences between the two groups using Prism version 7.03 (GraphPad Inc., San Diego, CA). *p*-value < 0.05 was considered statistically significant.

## Results

### Metformin Suppressed the Growth of Endometriotic Implant

Endometriosis was surgically induced using a Wistar albino mature rat endometriosis model. Rats were treated with either metformin (treatment Group 2-ML and Group 3 MH) or saline (Group 1, control). At the beginning of the treatment, the mean volumes of endometriotic implants were similar in the three groups of rats (Control: 65.19 mm^3^ ± 19.98, ML:79.35 ± 44.76, MH:80.90 mm^3^ ± 30.11; *p* > 0.05) (Table 1). At the end of day 21 post the initial operation, endometriosis implants in the abdominal wall were formed in 32 out of the 35 rats. At the end of the treatments, implant volumes were significantly lower in rats with both dosages of metformin treatment (Control: 198.39 mm^3^ ± 73.75, ML: 78.60 mm^3^ ± 25.11, MH: 117.18 mm^3^ ± 55.12; *p* < 0.001) (Table 1) with 100 mg/kg the optimal dosage. The overall volume of endometrial implants was larger in rats treated with 200 mg/kg of metformin (117.18 mm^3^ ± 55.12) as opposed to those treated with 100 mg/kg of metformin (78.60 mm^3^ ± 25.11). However, the difference in the two treatment groups was not significant (*p* = 0.152). A total of 27 rats with endometriosis completed treatment and five rats died (2 in each treatment group and 1 in the control group) during the period of treatment.

**TABLE 1:** Implant Volume and Histopathologic Score from three groups.

Variable	“Control Group Saline (*n* = 9)”	Metformin 100 mg (*n* = 9)	Metformin 200 mg (*n* = 9)	*p*-value^a^
Volume of implants (mm^3^)				
Before Treatment	65.19 ± 19.98	79.35 ± 44.76	80.90 ± 30.11	>0.05
After Treatment	198.39 ± 73.75	78.60 ± 25.11***	117.18 ± 55.12**	<0.001
Histopathologic Score of Implants on VEGF and MMP-9				
VEGF	5.11 ± 1.76	3.56 ± 0.88*	3.95 ± 1.35*^#^	<0.05
MMP-9	6.44 ± 1.33	4.22 ± 1.20**	4.89 ± 1.05*^#^	<0.01
Histopathologic Score of endometrium on LIF and HOXA10				
LIF	4.00 ± 1.41	5.56 ± 1.26*	4.55 ± 1.03^#^	<0.001
HOXA10	5.11 ± 1.45	6.89 ± 1.63*	6.00 ± 1.53^#^	<0.01

Data are presented as Mean ± SD. *p < 0.05, **p < 0.01 and ***p < 0.001 *versus* control group; ^#^ *p* < 0.05 when compared with ML, groups.

### Metformin Decreased VEGF and MMP-9 Expression in Rat Endometriotic Implants

To evaluate the effect of metformin on endometriotic implants in rats with endometriosis, VEGF and MMP-9 expression in implants were analyzed using IHC and western blotting.

**FIGURE 2:**
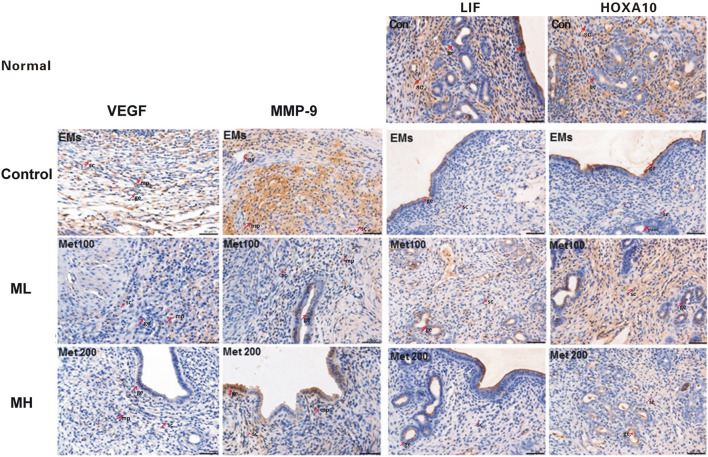
The effects of metformin on the expression of VEGF, MMP-9, LIF, and HOXA10. At the end of 6 weeks treatment, excised endometriotic implants from rats with endometriosis treated with either metformin (ML and MH groups) or saline (control group) were fixed, stained, and analyzed by immunohistochemistry (IHC). Images were representative of the effects of metformin on the expression of VEGF (3 images on the left); MMP-9 (3 images on the second column) in the endometriotic implants and LIF (images on the third column) and HOXA10 (3 images on the right column) from endometrium from rats with endometriosis. While VEGF immunopositive staining was mainly localized in glandular epithelial cells, vascular endothelium and surrounding stromal cells, as well as in the cytoplasm of macrophage cells, MMP9 expression was mainly located in the glandular epithelial cells, stromal cells and the macrophage cytoplasm, shown as pale to dark brown staining. IHC analysis revealed that metformin decreased VEGF and MMP-9 expression in rat endometriotic implants. LIF and HOXA10 expression, showing as brownish yellow to brown staining, was mainly located in the endometrial luminal epithelial, glandular epithelium and the cytoplasm of stromal cells. The H-score of LIF and HOXA10 was significantly increased in the endometrium from metformin treated rats when compared to the control. Red arrows in the images point to typical staining in glandular epithelium cell (ge), stromal cell (sc) and macrophage (mp). Scale bar = 50 µm.

As shown in Figure 2 left column, VEGF immunopositive staining was mainly localized in glandular epithelial cells, vascular endothelium and surrounding stromal cells, as well as in the cytoplasm of macrophage cells, shown as pale to dark brown staining. The percentage of positive stained cells and intensity of VEGF expression significantly reduced following metformin treatment. VEGF was weakly positive in macrophages and stromal cells and negative in the glands in the ML group. IHC analysis revealed that the VEGF H-scores of implants were significantly lower in metformin treated rats when compared to the control group (Control: 5.11 ± 1.76, ML:3.56 ± 10.88, MH:3.95 ± 1.35; *p* < 0.05) (Table 1; Figure 2).

MMP9 immunopositive staining was mainly located in the glandular epithelial cells, stromal cells and the macrophage cytoplasm, shown as pale to dark brown staining (second left column, Figure 2). After treatment, the percentage of positive stromal cells and macrophages significantly decreased, with fewer scattered brown staining in the ML group (Figure 2). Interestingly, in the MH group the intensity of MMP-9 positive cells were greater in the glandular epithelial cells compared to the ML group. The MMP-9 H-scores from the implants showed significant decrease in ML and MH groups when compared to the control group (Control: 6.44 ± 1.33, ML: 4.20 ± 1.20, MH: 4.89 ± 1.05; *p* < 0.01) (Table 1; Figure 2).

**FIGURE 3:**
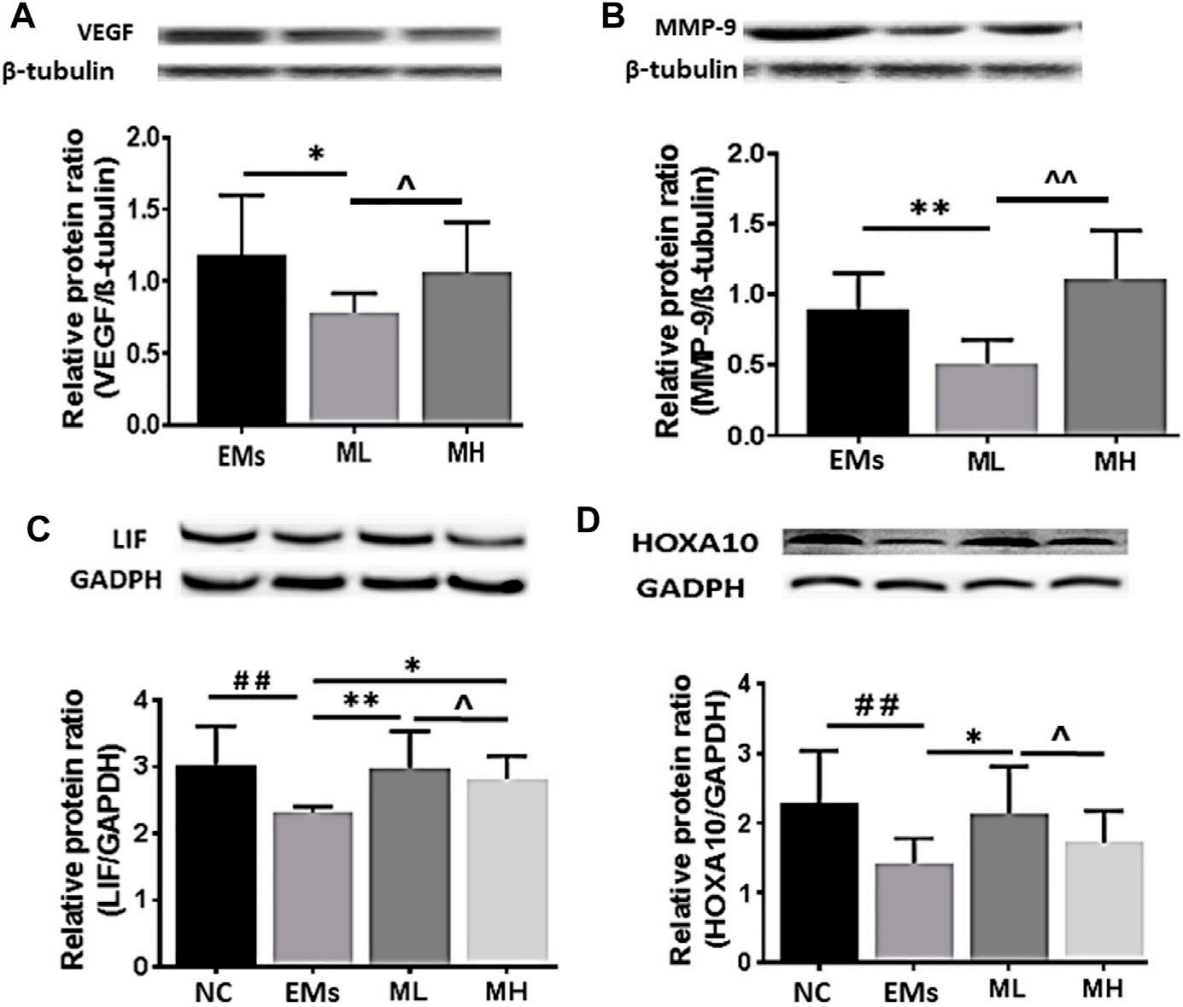
The protein expression of VEGF, MMP-9, LIF and HOCA10 in endometriotic implants. Representative Western blot images showed the effects of metformin on the expression of **(A)** VEGF and **(B)** MMP-9 in the implants and **(C)** LIF and (D) HOXA10 in the endometrium from rats with endometriosis. NC: normal control; EMs: endometriosis models; ML: metformin 100 mg/kg/d; MH: metformin 200 mg/kg/d. Experiments were performed in biological triplicates and histograms show the average ±SD. *p < 0.05 and **p < 0.01 treatment groups (ML or MH) versus EMs, ^##^ *p* < 0.01 normal control (NC) versus EMs; *^ p* < 0.05 ML versus MH.

Western blots showed significant reduction of VEGF expression in ML treated rats when compared to control (*p* < 0.05, Figure 3A), consistent with the IHC H-score. Interestingly, no significant difference in VEGF was observed between the MH and control group (*p* > 0.05, Figure 3A). Differences in VEGF between ML and MH groups were not significant (*p* > 0.05, Figure 3A). MMP-9 expression was significantly decreased in the ML group when compared to the control group (*p* < 0.01, Figure 3B). However, MMP-9 expression was not affected in the MH group when compared to the control group (*p* > 0.05, Figure 3B). Similarly, no significant difference in MMP-9 was observed between the ML and MH groups (*p* > 0.05, Figure 3B).

Taken together, these data suggest that metformin treatment significantly decreased VEGF and MMP-9 expression and the effect was optimal at the dose of 100 mg/kg.

### Metformin Increased LIF and HOXA10 Expression in Rat Endometrium During Endometriosis

In this study, IHC, Western blots and qRT-PCR were applied to determine the expression of two implantation markers, LIF and HOXA10, in the rat endometrium. IHC staining demonstrated that location of LIF and HOXA10 expression was mainly in the endometrial luminal epithelial, glandular epithelium and the cytoplasm of stromal cells, presenting as pale to dark brown staining (right 2 columns, Figure 2). In the control group LIF was expressed as weakly positive in the glandular epithelia and very weak or negative in the stromal cells. After treatment, the percentage of LIF positive staining in the glandular epithelia significantly increased in both ML and MH groups. The intensity of staining in the ML group was stronger than in the control and MH groups. LIF stained negatively in stromal cells in both the control group as well as in MH groups. The H-score of LIF was significantly increased by 39.0% in the endometrium from ML rats when compared to control (Control: 4.00 ± 1.41, ML: 5.56 ± 1.26, MH: 4.55 ± 1.03; *p* < 0.05) (Table 1; Figure 2). Likewise, there was a significant increase (34.8%) in the H-score of HOXA10 compared to the control group (Control: 5.11 ± 1.45, ML: 6.89 ± 1.63, MH: 6.00 ± 1.73; *p* < 0.05) (Table 1; Figure 2).

Using Western blot analysis, LIF expression was shown to be significantly higher in the endometrium resected from rats treated with both dosages of metformin than in rats from the control group (both *p* < 0.01, Figure 3C). Protein expression of HOXA10 was increased in the endometrium only from the MH group (*p* < 0.05, Figure 3D), whereas there was no significant difference in HOXA10 expression between the ML group and the control group (*p* > 0.05, Figure 3D).

**FIGURE 4:**
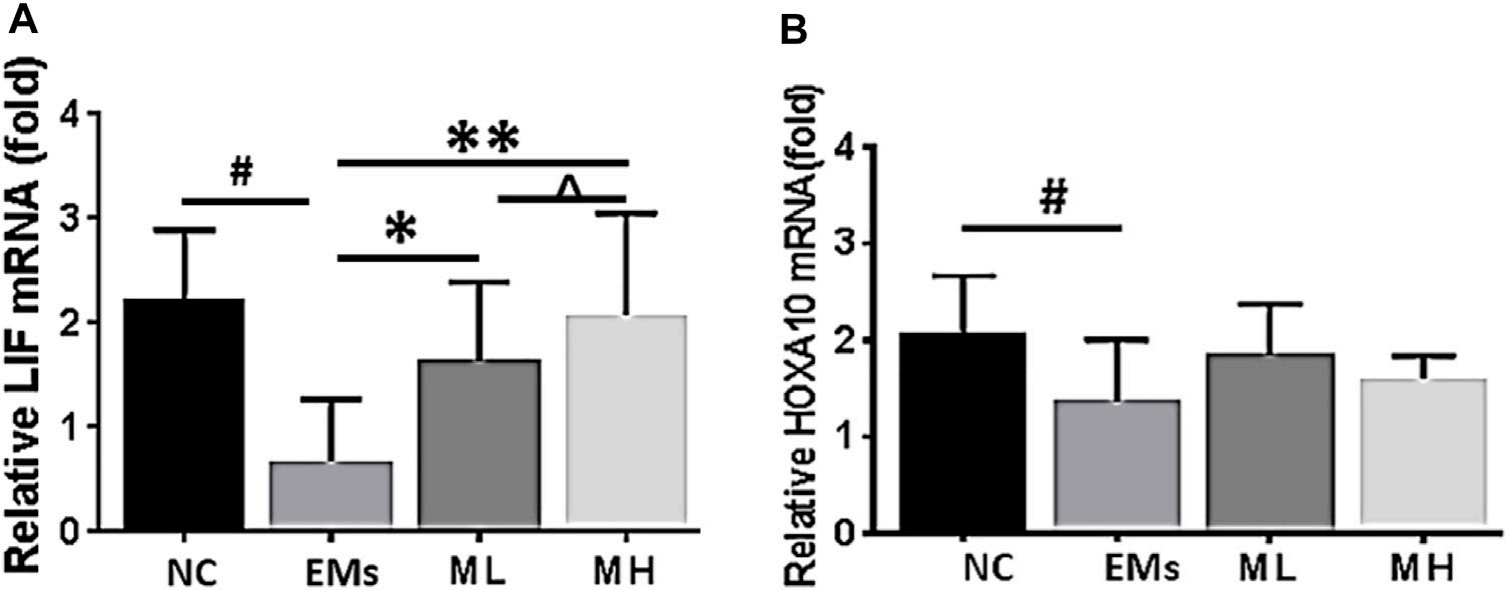
Effects of metformin on the expression of mRNA for LIF and HOXA10 in the endometrium from rats of endometriosis. Leukemia inhibitory factor (LIF) and Homeobox 10 (HOXA10) mRNA expression in the endometrium from rats with endometriosis. NC: normal control; EMs: endometriosis models; ML: metformin 100 mg/kg/d; MH: metformin 200 mg/kg/d. *p < 0.05 **p < 0.01 treatment groups (ML or MH) *versus* EMs group; ^#^ *p* < 0.05 normal control (NC) group versus EMs group; *^ p* < 0.05 ML group *versus* MH group.

To advance our understanding of the molecular mechanism by which metformin influences implantation markers, mRNA for LIF and HOXA10 were further investigated in this study. The mRNA fold change of LIF significantly increased in the MH treated rats compared to the control group (*p* < 0.001, Figure 4A). However, the level of HOXA10 mRNA in the ML and MH groups remained similar to the control group (*p* > 0.05, Figure 4B). Taken together, in the endometrium resected from rats with endometriosis, metformin increased LIF protein and mRNA expression, and enhanced HOXA10 protein expression dependent on metformin dosage.

## Discussion

Endometriosis is a key factor in reducing endometrial receptivity, thus impeding successful embryo implantation. Currently, there is a lack of effective medical treatment for women with endometriosis who are having problems conceiving. In the present *in vivo* study, we demonstrated that metformin treatment effectively alleviated endometriosis, as evidenced by reduction in the volume of endometriotic implants. We found that potential molecular mechanisms by which metformin regresses endometrial implants were associated with the inhibition of VEGF and MMP9. This study also revealed that improvement in endometrial receptivity in rats with endometriosis treated with metformin also correlated with an increase in the expression of the implantation markers, LIF and HOXA10, within the endometrium. Thus, the results of this study, together with previous reports (Oner et al., 2010; Yilmaz et al., 2010), provides strong evidence to include metformin as a feasible, potential option or adjunct therapy to optimize fertility for women of reproductive age with endometriosis.

Although the study on metformin for the treatment of endometriosis began in 2007 (Takemura et al., 2007), and a number of *in vitro* experimental studies (Zhang et al., 2015; Zhou et al., 2015)and *in vivo* rat animal model studies (Oner et al., 2010; Yilmaz et al., 2010), and one clinical study (Foda and Aal, 2012)on the use of metformin for the treatment of endometriosis, have been conducted since, little is known about the mechanism. The underlying mechanism by which metformin suppresses endometrial implants is thought to be associated with the inhibition of VEGF and MMP9 (Yilmaz et al., 2010). Previously VEGF inhibitors were shown to reduce the establishment, maintenance, and progression of endometriotic lesions in different laboratory and animal models of endometriosis (Machado et al., 2010; Ergenoğlu et al., 2013). However, clinical evidence for the efficacy and safety of VEGF inhibitors is lacking (Liu et al., 2016). In this study, VEGF expression in endometrial implants was significantly supressed with metformin treatment. Possibly VEGF suppression may subsequently reduce local blood supply to pathological tissues, leading to regression of endometrial implants. The correlation between an increase in MMP-9 expression and increase in endometriosis has been confirmed in clinical studies (Liu et al., 2015) and reduction in MMP-9 levels has been suggested as an important clinical biomarker for endometriosis treatment effectiveness (Machado et al., 2016; Kim et al., 2017). In keeping with this, we found that metformin treatment in our study significantly reduced MMP-9 expression accompanied with a decrease in the volume of endometrial implants from rats with endometriosis.

An important aspect of the current study was that we found that the inhibitory effect of metformin on endometriotic implants, VEGF and MMP9 was optimal at the dose of 100 mg/kg/d in preference to the higher dose of 200 mg/kg/d (Table 1 and Figure 2). Our *in vivo* studies showed that the decrease in the volume of endometriotic implants in the group receiving the dose of 100 mg/kg/d is more prominent than that in the higher dose group. Data from IHC and Western blot analysis also clearly demonstrated that the inhibition of metformin on VEGF and MMP-9 was stronger at the dose of 100 mg/kg compared with 200 mg/kg, meaning that higher dose demonstrated a weaker effect. Metformin has been shown to induce ovulation and affect female hormone levels in PCOS patients (Nestler, 2008; Sharpe et al., 2019). As previously demonstrated in a clinical trial, a higher dose of metformin led to improved ovulation rate and higher levels of estrogen in patients with PCOS (Ben Ayed et al., 2009). As estrogen levels are positively associated with the pathophysiology of endometriosis (Chantalat et al., 2020), the diminished therapeutic effect of increased metformin dosage observed in our study may be associated with the change in estrogen/female hormone levels related to the use of metformin. However, this is yet to be tested.

This study has demonstrated the beneficial effects of metformin in a rat model, leading to marked regressed of endometriotic implants associated with decreased expression of two markers biomarkers associated with endometrial receptivity. This effect was demonstrated after treatment with metformin for 6 weeks. However to prevent recurrence of endometriosis it is important to inhibit endometriotic implant growth meticulously (Selçuk and Bozdağ, 2013), and thus by increasing the duration of metformin treatment there is the possibility of complete growth inhibition of endometrial implants. A longer-term study may be able to further elucidate the favorable effects of metformin on endometriosis.

As a highlight of this study, we found that metformin treatment significantly increased the levels of both mRNA and protein for LIF. To our knowledge, this is the first report to demonstrate that metformin upregulates LIF expression in the endometrium during endometriosis. We believe that the beneficial effects of metformin in the treatment of endometriosis is a result of combination of diminishment endometrial lesions through suppression of VEGF and MMP9, and improvement of uterus conditions for implantation via LIF upregulation. Endometriosis is a primary cause of female infertility; the disease is frequently associated with impaired uterine receptivity, leading to implantation failure (Ulukus et al., 2006). Molecular mechanisms underlying the impaired uterine receptivity in endometriosis are mainly increased inflammatory cytokine and abnormal implantation markers (Cakmak and Taylor, 2011), with LIF and HOXA10 playing important roles in embryo implantation and development. Previous studies have suggested that LIF expression is essential to induce a receptive uterus for implantation (Stewart et al., 1992; Charnock-Jones et al., 1994; Schmitz et al., 2017), we therefore evaluated LIF mRNA and protein expression in the endometrium of rats treated with metformin.

In endometriosis, imbalance of sexual hormones with decreased progesterone and altered expression of the progesterone receptor leads to decreased expression of progesterone-responsive genes, including HOX genes in the eutopic endometrium. Reduction in HOX genes leads to downregulation of other mediators of endometrial receptivity involved in infertility associated with endometriosis, such as pinopodes, αvβ3 integrin, and IGFBP-1 (Cakmak and Taylor, 2010). In addition, other studies have demonstrated that metformin reduces the methylation levels of the peroxisome proliferator-activated receptor γ (PPAR γ) coactivator-1A of rat offspring with gestational diabetes mellitus (Song et al., 2016). In this study, HOXA10 protein expression was significantly increased in endometrium from rats treated with high doses of metformin. How metformin promotes the expression of HOXA10 has not been studied. It may occur through the regulation of PPAR γ pathway. Therefore, further investigation into this interaction would be important in future studies.

Endometriosis is an enigmatic, multifactorial disease and the use of rat implant models to mimic the human environment can be challenging. A number of factors may affect the growth of the endometriotic implants *in vivo* including expression of VEGF, MMP-9 as well as the persistent inflammatory environment of the pelvic cavity. However, functional differentiation of endometrium in uterine is normally regulated by oestrogen and progesterone (Niklaus et al., 2003). Women diagnosed with PCOS and endometriosis frequently exhibit irregular ovulation cycles and damaged corpus luteum function (Kocak et al., 2002; Abu Hashim et al., 2011; Sanchez et al., 2017), causing abnormal levels of oestrogen and progesterone. Metformin treatment has been shown to normalize oestrogen and progesterone levels by restoring normal ovarian function (Kocak et al., 2002; Mansfield et al., 2003; Abu Hashim et al., 2011; Maruthini et al., 2014). In this study, rats treated with high doses of metformin showed significantly increased expression of endometrial receptivity biomarkers (LIF and HOXA10), signifying that treatment with metformin might have improved corpus luteum function and receptivity. In fact, it was found in a clinical trial that metformin at a dose of 500 mg increased pregnancy rates in patients with endometriosis from 0 to 25.7% after 6 months treatment (Stochino-Loi et al., 2021).

Therapeutic effects of metformin on endometrium are multifold, including the enhancement of endometrial receptivity, improvement of vascularity, decrease of endometrial hyperplasia (Ayas et al., 2021), and reversal of atypical endometrial hyperplasia to normal endometrial histology (Meireles et al., 2017). Although the intricacies of the underlying mechanisms that lead to improvement of endometriosis are outside the scope of this study, our *in vivo* well-characterized rat endometriosis model makes for a good preclinical translational model for fast-track studies on this debilitating condition. Our data further demonstrates the potential of metformin, a commonly used FDA approved drug, as a promising candidate for endometriosis treatment. Our results clearly demonstrate the potentials of metformin in the treatment of endometriosis, in particular in the improvement of endometrial receptivity.

## Conclusion

In conclusion, the results of this animal study demonstrate that metformin alleviates endometriosis in rats via two separate mechanisms: 1) inhibiting angiogenesis and degradation of extracellular matrix to diminish the implants, 2) increasing LIF and HOXA10 expression in endometrium to improve endometrial receptivity in endometriosis. The effect of metformin on endometriosis in rats is maximised/optimal in this study at the dose of 100 mg/kg. Future translational studies are suggested to explore the possibility of metformin as a potential therapeutic agent in the treatment of endometriosis.

## Data Availability

The original contributions presented in the study are included in the article/Supplementary Material, further inquiries can be directed to the corresponding authors.

## Abbreviations

ANOVA, one-way analysis of variance; DAB, diaminobenzidine; FDA, food and drug administration; GnRH, gonadotropin-releasing hormone; IHC, immunohistochemistry; LIF, leukemia inhibitor factor; H&E, hematoxylin and eosin; HOXA10, homeobox A10; MH group, metformin high dose group (200 mg/kg/day); ML group, metformin low dose group (100 mg/kg/day); MMP-9, matrix metalloproteinase-9; PCOS, polycystic ovary syndrome; PVDF, polyvinylidene difluoride; qRT-PCR, quantitative real time polymerase chain reaction; SDS-PAGE, sodium dodecyl sulfate-polyacrylamide gel electrophoresis; TBS, tris buffered saline; VEGF, vascular endothelial growth factor.
